# Phenytoin-loaded bioactive nanoparticles for the treatment of diabetic pressure ulcers: formulation and in vitro/in vivo evaluation

**DOI:** 10.1007/s13346-022-01156-z

**Published:** 2022-04-11

**Authors:** Marwa M. Sheir, Maha M. A. Nasra, Ossama Y. Abdallah

**Affiliations:** Department of Pharmaceutics, Faculty of Pharmacy, Alexandria University, 1, El-Khartoum Square, Azarita, Alexandria, Egypt

**Keywords:** Chitosan, Alginate, Phenytoin, Diabetic pressure ulcers, Wound healing, Repurposing

## Abstract

Drug repurposing offers the chance to explore the full potential of existing drugs while reducing drug development time and costs. For instance, the anticonvulsant drug phenytoin (PHT) has been investigated for its wound healing properties. However, its poor solubility and variability of doses used topically limit its use. Hence, the aim of this study was to improve the properties and wound healing efficacy of PHT for the treatment of diabetic bedsores. PHT was encapsulated, using a modified ionic gelation method, in either positively or negatively charged chitosan-alginate nanoparticles (NPs), which possess previously demonstrated wound healing potential. These NPs were characterized by transmission electron microscopy, differential scanning calorimetry, and Fourier-transform infrared spectroscopy. PHT-loaded NPs were evaluated in vivo for their pressure ulcers’ healing potential using diabetic rats. The prepared NPs, especially the positively charged particles, exhibited superior wound healing efficacy compared to PHT suspension, with respect to healing rates, granulation tissue formation, tissue maturation, and collagen content. The positively charged NPs resulted in a 56.54% wound closure at day 7, compared to 37% for PHT suspension. Moreover, skin treated with these NPs showed a mature dermis structure with skin appendages, which were absent in all other groups, in addition to the highest collagen content of 63.65%. In conclusion, the use of a bioactive carrier enhanced the healing properties of PHT and allowed the use of relatively low doses of the drug. Our findings suggest that the prepared NPs offer an effective antibiotic-free delivery system for diabetic wound healing applications.

**Graphical Abstract:**

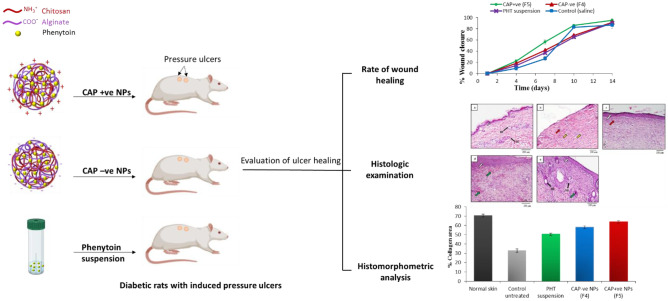

## Introduction

Diabetes is a fast-growing health problem affecting more than 463 million people worldwide, and this number is expected to rise [1, 2]. One of its major complications is poor and delayed wound healing, affecting about 15% of diabetic patients [3]. Chronic diabetic wounds develop as a result of several factors. For instance, sustained hyperglycemia results in high oxidative stress, the elevation of pro-inflammatory mediators, and the glycation of important proteins important for wound healing [4]. Moreover, diabetes is associated with vascular defects, poor angiogenesis, and impaired nerve function which might affect perception and lead to wound progression to worse. These factors eventually lead to immune cells and endothelial cells dysfunction, prolonged inflammation, delayed improper wound healing with increased liability to infection [3, 5]. Among chronic poorly healing wounds are pressure ulcers (PUs), also known as bedsores. These skin injuries result from prolonged pressure applied to certain bony areas such as the lower back, buttocks, hips, and heels. Hospitalization, wheelchair using or decreased mobility with age, together with diabetes-associated impaired microcirculation, are all risk factors for developing these ulcers. Prolonged application of pressure leads to impaired local blood flow, ischemia, and eventually tissue necrosis [6]. Although a number of wound dressings are available to help in the treatment of diabetic PUs, they are often not enough to ensure complete normal healing of these wounds, and additional therapies are usually necessary. Topical application of drugs is preferred over systemic administration to decrease possible side effects in non-target tissues and increase drug amount in the wound site, which is often poorly perfused in diabetics [3].

Increased understanding of the role of diabetes in impairing wound healing at a molecular level has broadened the wound healing research over the past years. For instance, a number of molecular factors have been identified which can directly or indirectly promote wound healing. For example, growth factors (e.g., vascular endothelial growth factor VEGF [7], platelet-derived growth factor PDGF [8]) have been found to increase angiogenesis, cell proliferation, and accelerate epithelialization in diabetic wounds in rats, compared to untreated ones. Autologous keratinocytes and stem cells can also directly enhance wound healing. On the other hand, various agents act indirectly by upregulating or downregulating the expression of some growth factors, enzymes, and cytokines [9]. Moreover, drug repurposing has also recently gained increased interest in several medical areas, including wound healing. Drug repurposing or repositioning is the identification and the use of approved drugs for new therapeutic indications not directly related to the original drug’s indication. This strategy offers a number of advantages over the development of an entirely new drug for a given indication. The most important advantages include shorter development time and relatively lower costs as most of the preclinical testing and safety evaluation have already been completed [10]. Phenytoin is an example of repositioned drug investigated for diabetic wound healing, which was found to promote collagen deposition and increase re-epithelialization [9, 11].

Phenytoin (PHT) was brought to the market in 1938 as an antiepileptic drug. Within the year after, gingival hyperplasia was reported as a side effect to PHT treatment, which led to the consideration of PHT in the treatment of gingival wounds [11]. Later on, several studies investigated its healing effect on various wounds including diabetic foot ulcers [12], pressure ulcers [13], venous ulcers [14], burns [15], traumatic wounds [16], and excisional wounds [17]. Numerous clinical studies investigated its wound healing effect on diabetic ulcers after topical application as a powder mixed with saline [12], aerosol powder [18] (Healosol^®^), cream [17], and liposomal-based lotion [14], and resulted in a significant reduction in healing time and accelerated formation of healthy granulation tissue. Its mechanism of action is thought to be due to the stimulation of fibroblast proliferation, increased collagen deposition, neovascularization, and expression of growth factors in the wounds [14, 17]. Although many studies, including clinical ones, have investigated the use of topical PHT for wound healing, the results are still controversial with a wide range of doses and dosage forms being used. For instance, among the clinical studies reporting a positive wound healing effect of PHT are Patil et al. [12], Muthukumarasamy et al. [19], and Pereira et al. [17] who used PHT suspension, powder, and cream, respectively, with different doses. In contrast, no significant healing outcomes were reported by Subbanna et al. [20] and Shaw et al. [21], who used PHT sodium-impregnated gauze and PHT-alginate dressing, respectively.

Phenytoin is practically insoluble in water. Its incorporation in a biocompatible nanocarrier would be beneficial in several aspects. First, reducing its particle size and increasing its surface area would improve its solubility. Also, the use of a nanocarrier helps control the release of the drug and facilitates the administration of controlled doses, all of which would improve its therapeutic efficiency at the wound site [22]. Additionally, the use of bioactive polymers as a delivery system contributes to the overall healing effect, thus allowing the use of lower doses of PHT.

To the best of our knowledge, only two studies have investigated the healing potential of PHT associated with nanocarriers [22, 23]. Teo et al. [22] reported that, using an in vitro cell monolayer scratch model with human adult keratinocytes, PHT-loaded alkyd nanoemulsions with 50 μg/mL PHT resulted in a significantly higher wound closure at 36 h, compared with the same concentration of PHT solution and control untreated cells. On the other hand, Cardoso et al. [23] investigated the wound healing effect of PHT when loaded in chitosan hydrogel either as free PHT, PHT in polycaprolactone nanocapsules or PHT nanoemulsion. They reported that PHT was able to increase the collagen content and fibroblasts in rats’ excisional wounds.

However, the effect of nano-encapsulated PHT on the treatment of diabetic wounds such as diabetic pressure ulcers has not been investigated so far. The use of bioactive polymers, such as chitosan and alginate which have intrinsic wound healing attributes [24, 25], for the encapsulation of PHT presents a promising approach for the treatment of diabetic PUs. Chitosan (CS) has been reported to have wound healing [24, 26] as well as an antibacterial effect [26–29]. Alginate (ALG) has also been reported to possess hemostatic and wound healing properties [30]. In our previous work [31], non-drug-loaded chitosan-alginate nanoparticles (CA NPs) showed a promising healing effect on both diabetic and non-diabetic PUs. Our main objective in this study was to investigate the wound healing potential of PHT combined with the bioactive wound healing chitosan-alginate NPs. This strategy would allow us to benefit from the advantages of the nanocarriers, as previously discussed, thus improving the properties of PHT and allowing for better interaction with the wound bed. In addition, the use of bioactive polymers, such as chitosan and alginate, wound enhance the wound healing properties of PHT and allow the use of relatively small doses of the drug.

## Materials and methods

### Materials

Chitosan (medium Mw: 100–300 kDa; deacetylation degree > 85%, viscosity of 1wt. % solution in 1% glacial acetic acid 20–50 cP) was purchased from Acros Organics™ (New Jersey, USA); sodium alginate Protanal CR8133 (M/G = 65/35, viscosity of 1 wt. % solution 20–40 cP) was obtained from FMC Health and Nutrition, Philadelphia, USA. Viscosities of both polymers were measured using cone and plate viscometer (Brookfield DV2T) at room temperature (25 °C). Phenytoin was acquired from El-Gomhouria Company for Drugs, Egypt. Isoflurane (Anahal^®^, from Pharco Pharmaceuticals, Egypt), Streptozotocin was obtained from Merck (Germany), glacial acetic acid, and all other reagents were analytical grade reagents.

### Preparation of phenytoin-loaded chitosan-alginate NPs (CAP NPs)

A modified ionic gelation method coupled with sonication [31] was used to prepare chitosan-alginate NPs with different ALG:CS weight ratios. Briefly, different concentrations of CS solution (0.05%, 0.075%, 0.1125%wt/v) were added dropwise to an equivalent volume of ALG solution (0.075%w/v), with stirring for 1 h (IKA Labortechnik, StaufenimBreisgau, Germany), followed by ultra-sonication for 15 min (Sonopuls HD 3100; BANDELIN, Berlin, Germany) and then stirring for 2 h. The NPs were then stored overnight in the refrigerator, then, separated by cooling centrifugation (40 min, 31,157 × *g*) (Model 3 K-30; Sigma Laborzentrifugen GmbH, Osterode, Germany). For PHT loading, the specified amount of PHT was first dissolved in 2 mL methanol (MeOH) and then added dropwise to ALG solution under stirring before adding CS solution.

The effect of adding different amounts of drug on the mean particle size, polydispersity index (PDI), and % entrapment efficiency (%EE) of CAP NPs was evaluated (F1-F3) using an ALG:CS weight ratio of 1:1. Therefore, different amounts of PHT were dissolved in 2 mL MeOH and added dropwise to ALG solution, followed by CS addition, to have different drug:polymer weight ratios (1:2.5; 1:5, and 1:10). Furthermore, the impact of using different ALG to CS weight ratios (1:0.67; 1:1, and 1:1.5) on NPs’ size, PDI, zeta potential (ZP), and %EE was investigated (F4, F2, and F5, respectively), at a fixed drug:polymer ratio of 1:5.

Five formulations were prepared, as shown in Table 1, where the polymers’ concentrations represent their initial concentrations prior to mixing.

**Table 1:** Composition of the different phenytoin-loaded chitosan-alginate naoparticles (CAP NPs) prepared

Formula code	ALG:CS weight ratio	ALG concentration ( %w/v)	CS concentration (%w/v)	Drug:polymer weight ratio
F1	1:1	0.075	0.075	1:10
F2	1:1	0.075	0.075	1:5
F3	1:1	0.075	0.075	1:2.5
F4	1:0.67	0.075	0.05	1:5
F5	1:1.5	0.075	0.1125	1:5

### Characterization of CAP NPs

#### Particle size, polydispersity index, and zeta potential measurements

Dynamic light scattering technique (Zetasizer Nano ZS, Malvern Instruments, UK) was used to determine the particle size (PS), polydispersity index (PDI), and zeta potential (ZP) of PHT-loaded NPs. The reconstituted CAP NPs were diluted with filtered deionized water. Measurements were performed in triplicates at 25 °C and the recorded values were represented as mean ± standard deviation (SD).

#### Determination of PHT entrapment efficiency

Percentage entrapment efficiency (%EE) of PHT in the NPs was calculated indirectly by determining the concentration of free un-entrapped PHT in the supernatant after separation of NPs by centrifugation. The supernatant was analyzed spectrophotometrically at a maximum wavelength of 258 nm (Cary 60 UV–Vis, Agilent, USA) [32]. All measurements were performed in triplicates.

The %EE was calculated as follows:

$$EE\,\%=\frac{Total\ PHT \,added-free\, PHT\, in\, supernatant}{Total\, PHT\, added}*100$$

#### In vitro drug release study

The in vitro release profile of PHT from different CAP NPs was evaluated using the dialysis bag diffusion method (VISKING^®^ dialysis tubing MWCO 12,000–14,000). Samples investigated included selected PHT-loaded CA NPs (F4 and F5), drug solution (in MeOH: H_2_O = 60:40), and drug suspension, where PHT was dispersed in deionized water. Samples corresponding to 1 mg PHT were filled in dialysis tubes and immersed in 15 mL 0.1 M phosphate buffer pH = 7.4 with 1% sodium lauryl sulfate (SLS) to obtain sink conditions and then placed in a shaking water bath at 32 °C, to simulate the in vivo skin conditions [33–35], at 100 rpm (JP Selecta Unitronic OR, Spain). The release medium was selected based on preliminary solubility studies to achieve sink conditions. At predetermined time intervals, 1-mL samples were withdrawn and compensated with the same volume of fresh release medium. The samples were analyzed using a UV spectrophotometer at 258 nm. All release experiments were run in triplicate and the values reported are the mean values ± SD.

#### Transmission electron microscope (TEM)

Morphology of the selected NPs (F4 and F5) was examined using transmission electron microscopy (Jeol JEM-1400 Plus, USA). For sample preparation, a drop of nanoparticles suspension was placed on a carbon-coated grid. After complete drying, the samples were stained with 2% w/v uranyl acetate.

#### Differential scanning calorimetry (DSC)

To prepare dry powdered CAP NPs, samples were redispersed in 5 mL deionized water, frozen, and then lyophilized for 24 h at – 55 °C and 0.5 mbar (Telstar, LyoQuest, USA).

Differential scanning calorimetry (DSC; Perkin Elmer Instruments, Waltham, MA, USA) was used to examine the thermal behavior of phenytoin-loaded NPs. Samples were hermetically sealed in aluminum pans and heated at a speed of 10 °C/min from 35 to 350 °C. The inert atmosphere was maintained by constant nitrogen purging at a rate of 20 mL/min. Samples included pure CS, ALG, PHT, and lyophilized PHT-loaded NPs (F4). The same conditions were applied to a control empty pan.

#### Fourier-transform infrared spectroscopy

Fourier-transform infrared (FT-IR) spectroscopy was performed to detect potential chemical interactions (Cary 630, Agilent, USA). Infrared spectra were scanned over the range of 400 to 4000 cm^−1^ using a resolution of 4 cm^−1^. Spectra of CS, ALG, PHT, their physical mixture, and selected PHT-loaded lyophilized CAP NPs (F4) were examined.

### Wound healing study

#### Animals

In vivo wound healing effect of the prepared PHT-loaded NPs was investigated in diabetic rats. All experiment protocols were approved by the Institutional Animal Care and Use Committee (IACUC), Faculty of Pharmacy, Alexandria University. Sixteen male Sprague Dawley rats, weighing 250–300 g were maintained at ambient temperature. Each of them was kept in a separate cage and was allowed food and water ad libitum.

#### Diabetic pressure ulcers’ healing study

**Fig. 1:**
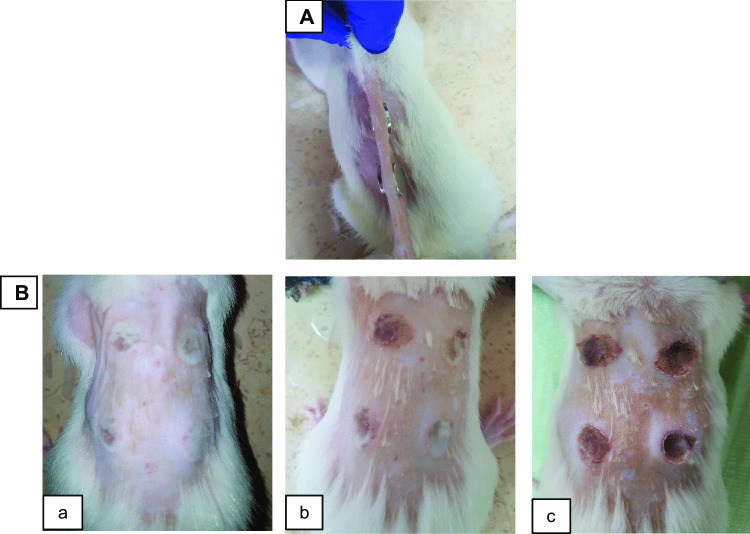
Photographs showing **A** the positioning of the magnets for pressure ulcers induction, **B** the progress of the formation of the ulcers after every ischemia–reperfusion cycle, (a) 1st cycle, (b) 2nd cycle, (c) 3rd cycle. The four ulcers were produced after three ischemia–reperfusion cycles

The diabetic pressure ulcer model was established as previously reported in our work [31]. Briefly, after fasting for 12 h, rats were injected intraperitoneally with one dose of Streptozotocin (STZ, 40 mg/kg body weight) dissolved in 0.5 mL 0.1 M citrate buffer (pH 4.5). Blood glucose levels (BGL) were measured before and three days after STZ injection. Diabetes induction is confirmed when a sustained hyperglycemic state is reached 48 to 72 h following STZ injection [36]. Rats with BGL of more than 250 mg/dL were selected for the study. Blood glucose levels were monitored throughout the study to ensure that the diabetic state was maintained. All rats maintained blood glucose levels > 500 mg/dL during the study. For pressure ulcers induction, diabetic rats were anesthetized by isoflurane inhalation. The back of each rat was then shaved and disinfected, and four ulcers were created using ischemia–reperfusion injury. The skin was gently pulled between 2 round neodymium magnets (diameter 12 mm, thickness 2 mm) [37]; 2 sets were used to produce 4 wounds on the back of each rat, as shown in Fig. 1A. Four pressure ulcers were formed, with a diameter of ≈ 12 mm, after three ischemia–reperfusion cycles. The stages of the development of these ulcers are shown in Fig. 1B. Each cycle consisted of a 12-h ischemia period, by magnets placements, and a 12-h reperfusion period, by magnets removal. The procedure seemed to be well tolerated by the rats as they resumed their usual activity few minutes after the placement of the magnets.

#### Treatment application

The healing effect of two PHT-loaded CA NP formulations was evaluated: negatively charged CAP NPs with excess alginate (F4), designated as CAP − ve, and positively charged CAP NPs with excess chitosan (F5), designated as CAP + ve. These formulas were compared to PHT suspension and control untreated groups. For the treatment of the induced pressure ulcers, PHT-loaded CA NPs were reconstituted in a small amount of deionized water, resulting in a viscous suspension with a final drug concentration of 5 mg/mL. PHT suspension was prepared in 1% HPMC. The daily dose consisted of 200 μL of NP suspension or drug suspension equivalent to 1 mg PHT. For the control group, the ulcers were cleaned daily with saline. The sixteen rats were divided into 4 groups, each group receiving one of the stated treatments and a group was kept as control. Treatments were applied, once daily for 14 days.

#### Evaluation of ulcer healing

Ulcers were digitally photographed every 3 days throughout the 14-day study period. The areas of the wounds were calculated using the image analysis software (ImageJ, National Institutes of Health, USA). The percentage of wound closure for each wound was calculated according to the following equation:

$$\%\,Wound\, closure=\frac{Initial\, wound \, area\,-Wound \,area\, remaining}{\,Initial \,wound\, area}\,\times\, 100$$

#### Histological and histomorphometric analysis

At day 14, rats were sacrificed and full-thickness skin biopsies of the ulcers with surrounding tissues were collected. Skin biopsies were fixed in 10% formalin solution and then embedded in paraffin. Next, 5-μm slices were cut and stained with hematoxylin and eosin stain and Masson’s trichrome stain. Examination of the slides for re-epithelialization, presence of inflammatory cells, and formation of skin appendages was carried out using a light microscope. Histomorphometric analysis was used to calculate the total area occupied by collagen fibers in the wound biopsies. When skin biopsies are stained with Masson’s trichrome stain, the blue color represents the collagen fibers. The slides were examined and photographed, and the percentage of blue staining in the total area of each image was evaluated using the color deconvolution software ImageJ. The morphometric analysis of the blue color was calculated as a percentage of total pixels in each image, using ImageJ software [38].

### Statistical analysis

One-way analysis of variance ANOVA was conducted for statistical analysis of data using SPSS Statistical Package 23.0 (SPSS Inc., Chicago, USA). The Tukey post hoc test for pairwise comparisons was applied when required. A *P*-value of ≤ 0.05 was considered statistically significant.

## Results and discussion

### Preparation of PHT-loaded chitosan-alginate nanoparticles

In an effort to improve PHT poor aqueous solubility, the hydrophobic drug was incorporated into the NPs after being dissolved in methanol prior to its addition to the ALG solution [39]. After the addition of CS, the two polymers formed discrete NPs which simultaneously entrapped the suspended PHT within their neutralized core. It is believed that, although each individual polymer is a hydrophilic charged polymer, the interaction between oppositely charged polyelectrolytes leads to the formation of particles which consist of a neutralized, relatively compact core, surrounded by a shell of the excess polymer, stabilizing the formed particles by electrostatic repulsion [40, 41]. Therefore, the end product consisted of a colloidal dispersion of CA NPs entrapping PHT. It is worth mentioning that chitosan and alginate polymers were selected in this study because, in addition to their intrinsic wound healing properties, the interaction between their opposite charges formed particles with a neutralized uncharged core which helped increasing the entrapment efficiency of an uncharged hydrophobic drug such as PHT.

#### Effect of drug loading

Table 2 shows the effect of adding different amounts of PHT on the PS, PDI, and %EE of CAP NPs prepared with ALG:CS weight ratio 1:1, where the weight of the polymer blend equals 22.5 mg. Increasing the PHT amount from 2.25 to 4.5 mg showed no significant increase in both PS and PDI (*P* > 0.05). On the contrary, the loading of 9 mg (F3) resulted in a significant increase in both PS and PDI (*P* < 0.001). The incorporation of PHT in the chitosan-alginate nanoparticles may have caused some reorientation of the polymers that form the nanosystem leading to a relative increase in size. This may be attributed to the interaction between PHT and the polymers which occurred at the expense of CS-ALG interactions, thus altering the way the two polymers associate [42]. Increasing the PHT amount to 9 mg might have led to a further alteration in the formation of the nanosystem, leading to a dramatic increase in particle size and the formation of a less compact system. A similar finding was observed with Goycoolea et al. [42] where insulin-loaded chitosan-alginate NPs showed a significantly higher PS than the blank NPs. In addition, Thai et al. [43] also observed a similar increase in losartan-loaded chitosan-alginate nanoparticles’ size when increasing the drug concentration, accompanied by a broader size distribution which might be due to strong drug-drug interaction at high drug concentration that may lead to the formation of drug agglomerates within the nanoparticles, thus causing an increase in the PS and PDI [43].

Concerning %EE, statistical analysis showed that there was no significant difference between formulas F2 (4.5 mg) and F3 (9 mg), while F1 (2.25 mg) showed a significantly lower %EE when compared to F2 and F3. A similar finding was observed by Das et al. [44] who reported that increasing the initial concentration of curcumin resulted in an increase in %EE. A possible explanation of this phenomenon may be as follows: increasing the initial amount of PHT led to an increase in the amount of suspended PHT which would rather be encapsulated in the neutral uncharged environment of the NPs’ core rather than in the aqueous outer environment.

From the previous results, it could be concluded that loading PHT with a drug:polymer weight ratio of 1:5 was the optimum selection for further investigations. This ratio resulted in a high %EE, together with lower particle size and more homogenous particle size distribution than the 1:2.5 ratio.

#### Effect of alginate to chitosan weight ratio

Different CS concentrations, thus different ALG:CS weight ratios were used in the preparation of PHT-loaded CA NPs. The calculated amount of PHT was dissolved in MeOH and added dropwise to ALG solution, to obtain a drug:polymer ratio of 1:5. Then, a fixed volume of CS (15 mL) with different concentrations (0.05%, 0.075%, 0.1125%) was added dropwise to ALG solution yielding different ALG:CS weight ratios (1:0.67, 1:1, 1:1.5 respectively). As illustrated in Table 2, PS increased significantly when CS concentration was increased from 0.05% (F4) to 0.075% (F2) and 0.1125% (F5) (*P* < 0.05). On the contrary, there was no significant increase in PS between F2 and F5. The increase in PS at higher CS concentration may be due to the repulsion between the positive charges of the added CS, as previously reported by Mukhopadhyay et al. [45]. The PDI of PHT-loaded CA NPs was not significantly altered by the increase in CS concentration (*P* > 0.05).

Regarding the drug entrapment, F4 with the lowest CS concentration showed the highest %EE of 90 ± 1.5%, which was significantly higher than the other formulations. Increasing CS concentration led to a decrease in %EE. Similar findings were observed with Motwani et al. [46], where the entrapment of gatifloxacin in chitosan-alginate nanoparticles decreased with increasing polymers concentration. A possible explanation is that at high polymers concentrations, the polymers make the bulk of the nanoparticles’ matrix and less volume is available for drug encapsulation [46].

**Table 2:** Effect of the variation of the amounts of drug added and ALG: CS weight ratio on particle size (PS), polydispersity index (PDI), zeta potential (ZP), and percent entrapment efficiency (%EE) of the prepared phenytoin-loaded chitosan-alginate nanoparticles (*n* = 3)

Formula code	Drug:polymer weight ratio (PHT weight)	ALG:CS weight ratio	PS (nm) ± SD	PDI ± SD	ZP (mV) ± SD	%EE ± SD
**F1**	1:10 (2.25 mg)	1:1	317.9 ± 9.9	0.288 ± 0.040	43.2 ± 0.8	57.2 ± 2.0
**F2**	1:5 (4.5 mg)	1:1	366.5 ± 12.1	0.344 ± 0.087	46.7 ± 0.5	83.7 ± 6.1
**F3**	1:2.5 (9 mg)	1:1	492.8 ± 58.2	0.560 ± 0.051	43.2 ± 2.4	87.6 ± 3.0
**F4**	1:5 (4.5 mg)	1:0.67	208.8 ± 4.3	0.267 ± 0.016	− 34.6 ± 0.7	90.3 ± 1.5
**F5**	1:5 (4.5 mg)	1:1.5	366.5 ± 4.6	0.244 ± 0.020	51.5 ± 0.3	76.7 ± 0.9

### In vitro drug release study

The dialysis bag method was used to compare the release behavior of the PHT-loaded NPs to that of pure drug. For the release study, 2 formulas were selected, namely F4 and F5, to evaluate the influence of the presence of either excess chitosan or alginate on the release behavior of PHT from CA NPs. These formulations were compared to PHT suspension and PHT solution (in MeOH:H_2_O = 60:40).

**Fig. 2:**
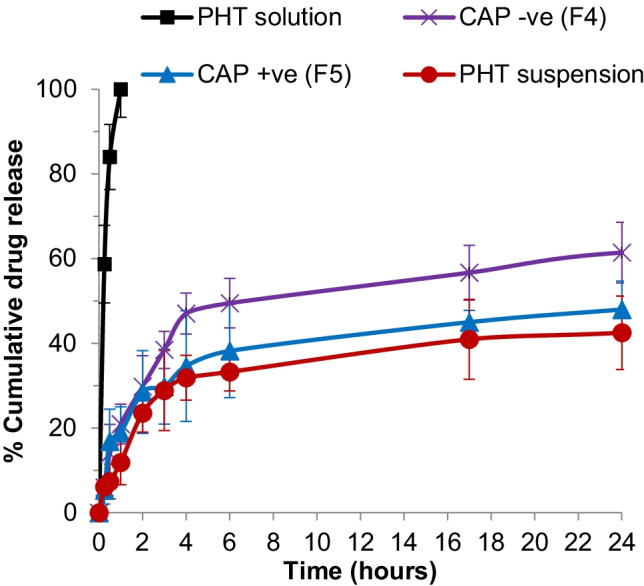
In vitro release profile of phenytoin from PHT-loaded chitosan-alginate nanoparticles compared to PHT suspension and PHT solution at 100 rpm and 32 °C in 0.1-M phosphate buffer pH7.4 + 1% SLS for 24 h, *n* = 3. PHT, phenytoin; CAP − ve, negatively charged chitosan-alginate nanoparticles; CAP + ve, positively charged chitosan-alginate nanoparticles

As shown in Fig. 2, complete release of PHT from solution occurred after 1 h, indicating the good dialysability of the drug.

The release of PHT suspension was relatively slower than the formulations, with 42% released after 24 h. Since PHT is a biopharmaceutical classification system (BCS) class II drug, the slow and incomplete dissolution and release of its crystalline powder can be expected and may be attributed to its hydrophobic nature, poor wettability, and particles agglomeration during the experiment. A negligible dissolution profile of crystalline PHT in phosphate-buffered saline of pH 6.5 containing simulated fasted duodenal solution was also observed with Widanapathirana et al. [47].

Regarding the nanosystems, a burst release was observed after 4 h for both formulas followed by a slow modulated release. A similar result was observed by Motwani et al. [46] during the release of gatifloxacin from chitosan-alginate NPs. This initial rapid release may be due to the rapid hydration of the NPs due to the hydrophilic nature of chitosan and alginate. The initially released PHT is most probably present on the surface or near the surface of the NPs [46, 48].

Both CAP NPs exhibited slightly enhanced extent and rate of release, compared to PHT suspension, with 48% and 61% released after 24 h for CAP + ve and CAP − ve, respectively. The enhanced dissolution rate may be explained by the smaller particle size of nano-sized PHT, thus a higher surface area available for dissolution [49]. When comparing both formulations, it was observed that CAP + ve NPs (F5) showed a relatively slower release than CAP − ve NPs (F4). The cumulative percentage release of PHT from CAP + ve NPs and CAP − ve NPs was 48% and 61% after 24 h. The difference in the release profiles could be attributed to the higher concentration of CS in CAP + ve which may retard the release of PHT from the NPs due to the poor solubility of CS in neutral conditions [50]. Similar results were obtained with Mukhopadhyay et al. [45, 51].

In general, both CAP NPs showed a sustained release pattern, compared to the PHT solution, which would be suitable for topical in vivo applications. In addition, the release of PHT from the CAP NPs indicates that the drug is not highly bound to the system and can be released from the nanoparticles.

### Physicochemical characterization

#### Transmission electron microscope

**Fig. 3:**
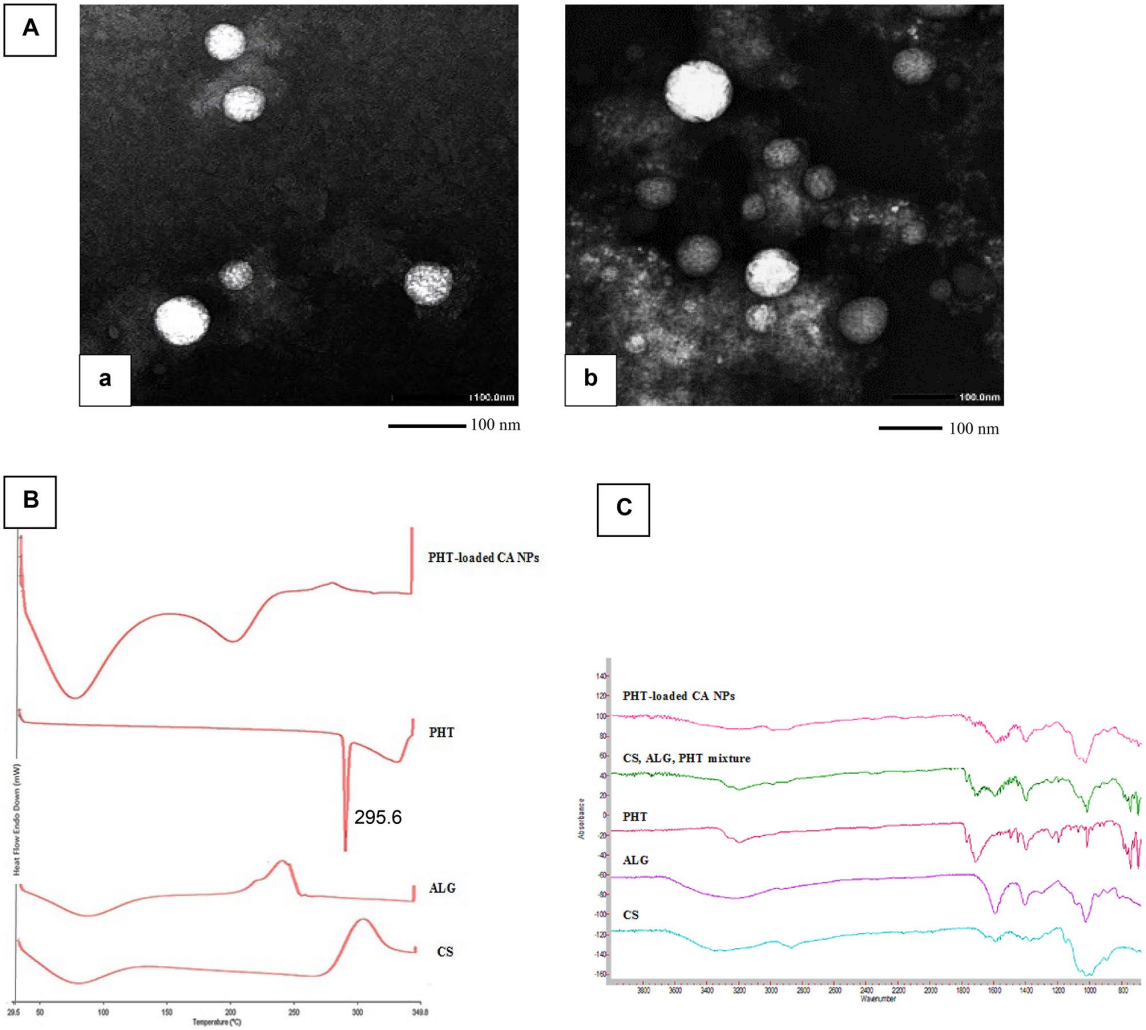
**A **Transmission electron microscopy images of phenytoin-loaded chitosan-alginate nanoparticles (a) CAP − ve NPs, negatively charged chitosan-alginate nanoparticles (b) CAP + ve NPs, positively charged chitosan-alginate nanoparticles, stained with uranyl acetate (scale bar = 100 nm); **B** differential scanning calorimetry thermograms of pure CS, ALG, PHT, and PHT-loaded CA NPs; **C** Fourier-transform infrared spectra for pure CS, ALG, PHT, their physical mixture, and PHT-loaded CA NPs. CS, chitosan; ALG, alginate; PHT, phenytoin; PHT-loaded CA NPs, phenytoin-loaded chitosan-alginate nanoparticles

Morphological examination of PHT-loaded negatively charged CAP NPs ( F4, named CAP -ve) and positively charged CAP NPs (F5, named CAP + ve), confirmed the formation of NPs with spherical shape in the nanometric range, as shown in Fig. 3A.

#### Differential scanning calorimetry (DSC)

DSC analysis was carried out to study any changes in the drug crystallinity. DSC thermogram of PHT showed a sharp endothermic peak at 295.6 °C related to its melting point which indicates the crystalline nature of the drug [32]. However, the thermogram of PHT-loaded NPs presented no peak in this region as shown in Fig. 3B. These results suggest that PHT was incorporated in CA NPs and that the crystalline nature of PHT may have been converted to the amorphous form [52, 53].

#### Fourier-transform infrared spectroscopy

FT-IR spectroscopy was carried out to characterize possible interactions between PHT and the polymers in CA NPs. The IR spectrum of pure PHT (Fig. 3C) showed characteristic absorption bands at 3195 cm^−1^ (N–H stretching), 1770 cm^−1^ (C = O stretching), and 744 cm^−1^ (phenyl ring C-H out of plane vibration). Similar findings were observed by Ali et al. [32]. On the other hand, the spectrum of PHT-loaded CA NPs showed the presence of PHT bands at 1772 and 744 cm^−1^, which confirms the successful entrapment of PHT in CA NPs. However, the characteristic absorption band of PHT at 3195 cm^−1^ disappeared. A similar finding was observed with Motawea et al. [53] which may be attributed to the interaction of the drug with the polymers, probably via intermolecular hydrogen bonding, which reflects drug entrapment inside the polymeric matrix.

### Wound healing study

#### Evaluation of ulcer healing

**Fig. 4:**
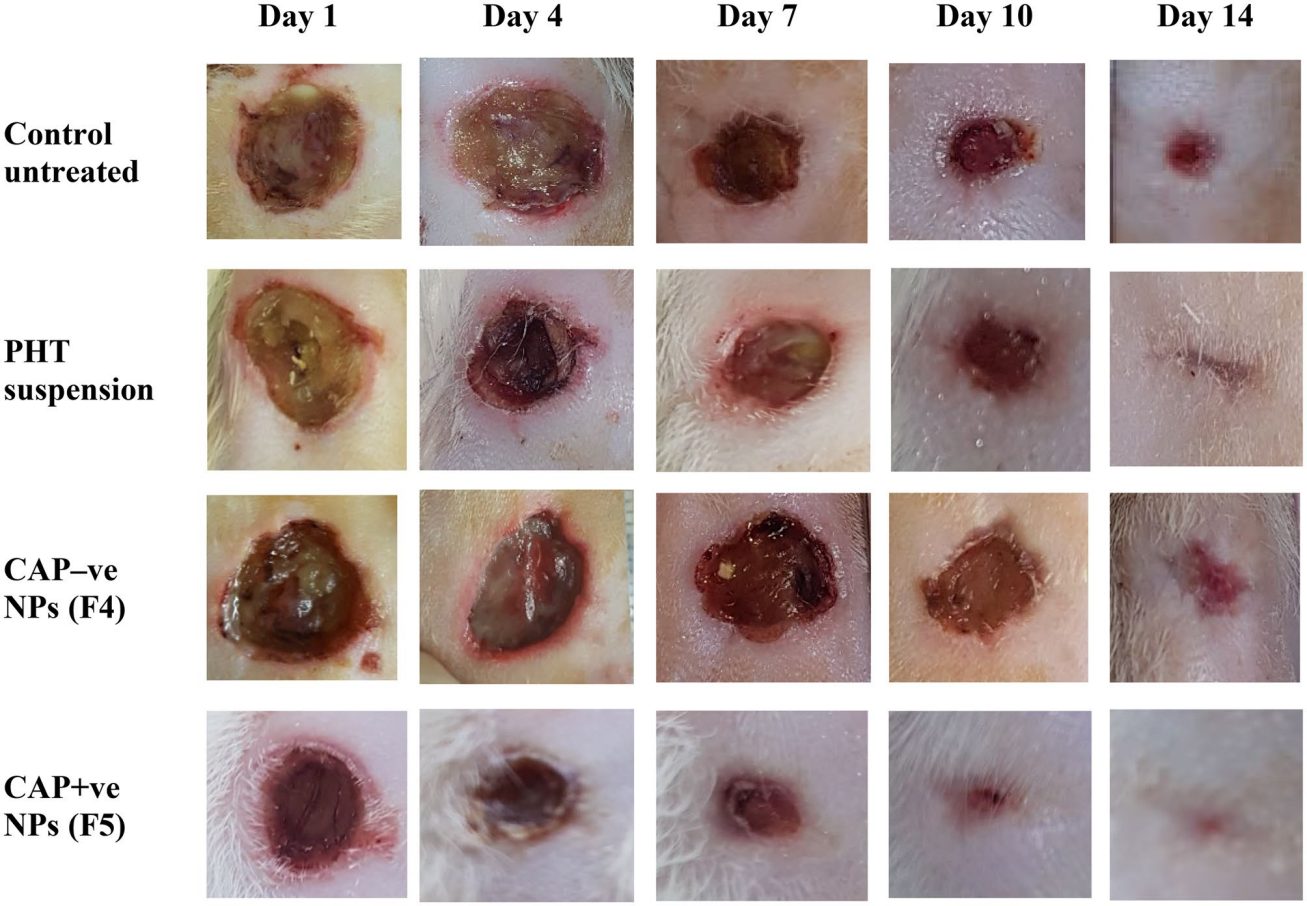
Stages of ulcers healing with the different treatments for 14 days. PHT, phenytoin; CAP − ve NPs, negatively charged chitosan-alginate nanoparticles; CAP + ve NPs, positively charged chitosan-alginate nanoparticles

**Fig.5:**
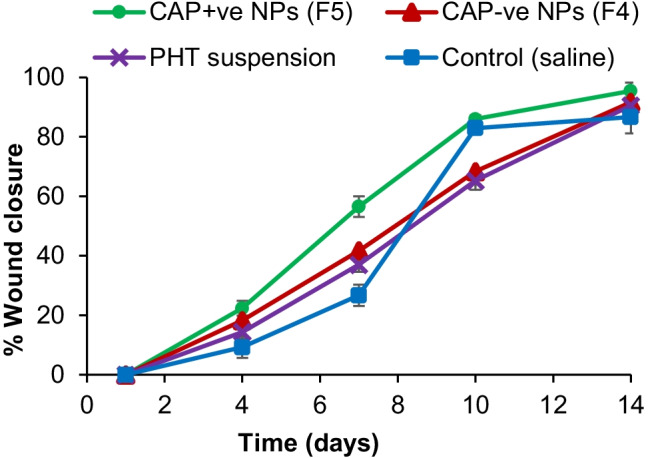
Effect of different treatments on percent wound closure of pressure ulcers of rats during 14 days, data are expressed as the mean ± standard deviation (*n* = 4 rats with a total of 16 wounds per group), PHT, phenytoin; CAP − ve NPs, negatively charged chitosan-alginate nanoparticles; CAP + ve NPs, positively charged chitosan-alginate nanoparticles

Diabetes induction is confirmed when a sustained hyperglycemic state is reached 48 to 72 h following STZ injection [36]. All rats maintained blood glucose levels > 500 mg/dL during the study. Ulcers were digitally photographed every 3 days until the end of the 14-day study. Figure 4 shows the progression of the healing of the ulcers throughout the experiment. All ulcers exhibited progressive healing; however, healing rates were different. PHT-loaded NPs and PHT suspension showed significantly higher initial healing rates over the first two time intervals (4 and 7 days) compared to the control group (*p* < 0.05), as shown in Fig. 5. For instance, at day 4, PHT suspension (14.35%) significantly improved the percentage of wound closure compared to the control group (9.24%), followed by the negatively charged NPs (18.25%) and finally the positively charged NPs (22.38%) which showed the highest percentage of wound closure. This observation persisted at day 7, with the positively charged NPs resulting in a significantly higher percentage of wound closure of 56.54% compared to the other groups. It is worth mentioning that PHT-loaded nanoparticles have not been assessed so far for the treatment of diabetic pressure ulcers. However, several clinical studies investigated the use of PHT for the treatment of various wounds and reported faster healing time and more epithelialization in wounds treated with PHT compared to the control group. Among these, reports were Patil et al. [12] who used 100 mg PHT powder mixed with saline for 0–5 cm^2^ diabetic foot ulcers [12] and Pereira et al. [17] who studied the use of PHT cream 0.5% for excisional wounds on the face and on the back [17], but did not determine the exact daily dose used. Hokkam et al. [14] also reported a shorter healing time of chronic venous ulcers in patients treated with a lotion consisting of 1 g PHT mixed with 25 mL liposomal base, compared to saline. These results are consistent with ours regarding the shorter healing time for PHT-treated groups, compared to control groups. In our study, the daily dose used was 1 mg PHT per ulcer, which had an initial diameter of about 1.2 cm, hence an area of about 1.13 cm^2^. The positive healing effects obtained with the use of such a low dose suggest the promising effect of using PHT in a nanosystem.

#### Histological analysis

**Fig. 6:**
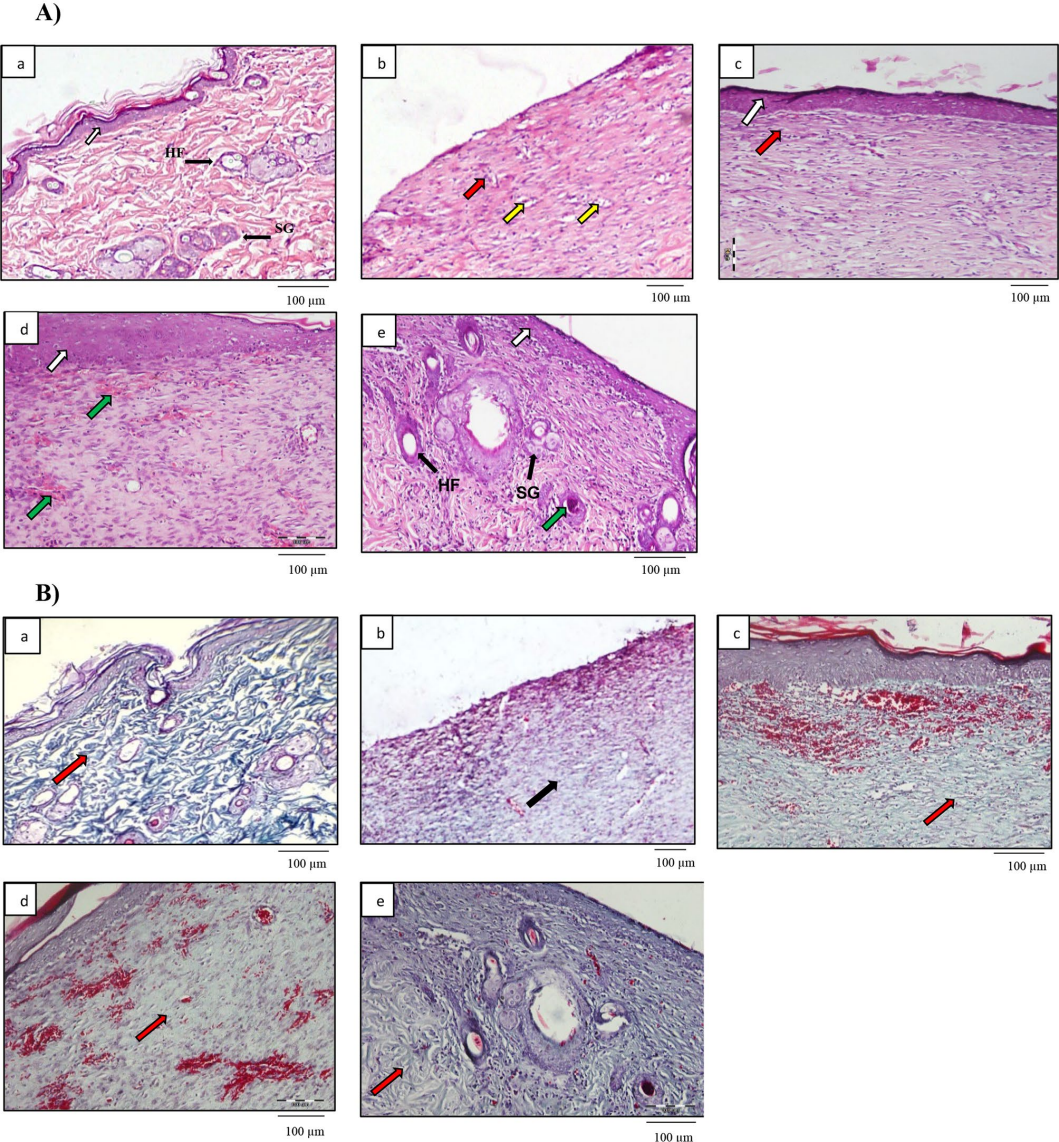
**A **Histologic examination using hematoxylin and eosin (H&E) stain of (a) normal skin, (b) control untreated group, (c) treated with PHT suspension, (d) treated with CAP − ve NPs (e) treated with CAP + ve NPs, showing epithelium (white arrow), inflammatory cells (red arrow), edema (yellow arrow), new blood vessels (green arrow), skin appendages (black arrow), HF, hair follicle; SG, sebaceous gland; **B** histologic examination using Masson’s trichrome stain of (a) normal skin, (b) control untreated group, (c) treated with PHT suspension, (d) treated with CAP − ve NPs, (e) treated with CAP + ve NPs, showing normal random collagen arrangement (red arrow) and fibrosis with parallel condensed collagen bundles (black arrow). PHT, phenytoin; CAP − ve NPs, negatively charged chitosan-alginate nanoparticles; CAP + ve NPs, positively charged chitosan-alginate nanoparticles

Figure 6A shows the hematoxylin and eosin (H&E)-stained images of the ulcers treated with the different therapies throughout the study.

The results showed that PHT had a great impact on improving the quality of regenerated skin. All PHT-treated groups showed well-structured regenerated epithelium and significantly less inflammation compared to the control untreated group (Fig. 6A (b)). Moreover, the incorporation of PHT in CA NPs resulted in improved skin quality and maturity. For instance, in addition to the re-epithelialization and mild inflammation seen in the PHT suspension group (Fig. 6A (c)), the CAP − ve NP group (Fig. 6A (d)) showed well-organized dermis with marked neovascularization. On the other hand, remodeling of the epidermis and dermis was more mature in the CAP + ve NPs group (Fig. 6A (e)) compared to the other groups. Well-organized dermis structure similar to the normal structure, in addition to the formation of skin appendages, such as hair follicles and sebaceous glands, was also observed in this group. Similar findings were reported by Cardoso et al. [23] who studied the wound healing effect of chitosan hydrogels loaded with either free PHT, PHT in polycaprolactone nanocapsules or PHT nanoemulsion at 0.025% w/v, for the treatment of excisional wounds in rats, over 6 days. They reported that PHT-treated groups showed no necrotic foci, less inflammation, and evident collagen fibers and fibroblast proliferation, compared with the control group and groups treated with unloaded nanocarriers. PHT has been reported to promote wound healing by multiple mechanisms. First, it promotes the maturation of collagen in normal and granulation tissue. In addition, PHT was reported to enhance neovascularization [54]. The above results suggest that our system resulted in favorable wound healing rates and quality of the formed skin while using as low as 1 mg PHT daily for 14 days. This might be attributed to its use as a nano-sized system with a bioactive carrier. It is worth mentioning that PHT-loaded NPs, both CAP − ve and CAP + ve, resulted in less inflamed and more mature skin structure, compared to their corresponding blank CA NPs reported in our previous work [31], suggesting that the incorporation of PHT enhanced the wound healing properties of the chitosan-alginate NPs.

#### Histomorphometric analysis of collagen

During granulation tissue formation in the wounded area, fibroblasts synthesize the new extracellular matrix of which collagen is the main component, thus replacing the provisional matrix consisting initially of fibrin [38]. Figure 6B shows collagen fiber-stained blue with Masson’s trichrome stain after 14 days. Regarding the arrangement of collagen fibers, groups treated with PHT, shown in Fig. 6B (c, d, and e), showed a better organized and distributed collagen fibers compared to the control group shown in Fig. 6B (b).

**Fig. 7:**
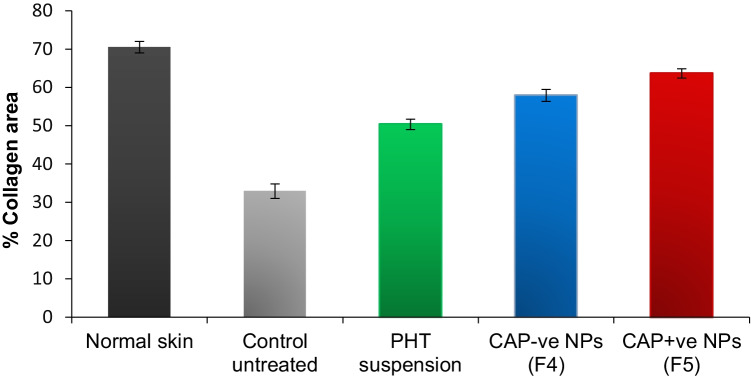
Quantification of collagen formation in wound samples of diabetic rats receiving various treatments, on day 14, data are expressed as the mean ± standard deviation (*n* = 4 rats with a total of 16 wounds per group), PHT, phenytoin; CAP − ve NPs, negatively charged chitosan-alginate nanoparticles; CAP + ve NPs, positively charged chitosan-alginate nanoparticles

For each group, four slides were examined and four images per slide were captured to calculate the percentage of blue staining. The percent areas occupied by collagen fibers in the different groups are shown in Fig. 7. Treatment with PHT suspension (50.35 ± 1.34%) caused a significant increase in collagen amount compared to the control untreated group (32.9 ± 1.9%). Incorporation of PHT in CA NPs resulted in a further improvement in the deposition and organization of collagen fibers with % collagen areas of 63.65 ± 1.2% and 57.9 ± 1.55% for CAP + ve and CAP − ve, respectively, which were significantly higher than the other groups. Many previous studies as Habibipour et al. [54] and Cardoso et al. [23] also reported that topical PHT application caused an increase in collagen deposition compared to control groups, possibly by enhancing collagen cross-linking and inhibiting collagenases activity [54].

When comparing these results with the results of the blank chitosan-alginate nanoparticles in our previous study [31], it is worth mentioning that although PHT-loaded CA NPs did not produce a significant difference in the percentage of wound closure compared to the blank NPs, the addition of PHT significantly enhanced collagen deposition and improved the quality of the newly formed skin compared to blank NPs. Therefore, when evaluating a drug for wound healing, a better judgment of its therapeutic potential would be achieved when healing rates are complemented with histological examination.

## Conclusion

To the best of our knowledge, this is the first study to evaluate the wound healing potential of phenytoin when encapsulated in bioactive nanoparticles, such as chitosan-alginate nanoparticles. This system gave promising diabetic pressure ulcers’ healing results, which might be attributed to the use of polymers with intrinsic wound healing properties, allowing the use of as low as 1 mg phenytoin daily. Phenytoin was successfully encapsulated in both positively and negatively charged chitosan-alginate nanoparticles without the need for surfactants. Better healing rates and quality of the formed skin were obtained by increasing chitosan concentration, showing more mature skin with hair follicles, sebaceous glands, and blood vessels. The results of this study suggest that using a drug-bioactive polymer combination would result in a superior wound healing effect, compared to using either constituent alone, thus benefiting from the combination of different mechanisms of action and allowing the use of relatively low doses of the drug. We expect that this antibiotic-free system would contribute to advancements in the wound healing field. Future studies would evaluate the wound healing potential of these nanoparticles when incorporated in a suitable final dosage form, which would not interfere with their wound healing potential.

## Data Availability

All data generated or analyzed during this study are included in this published article.
